# How Do Family Physicians Perceive Their Role in Providing Pre-exposure Prophylaxis for HIV Prevention?–An Online Qualitative Study in Flanders, Belgium

**DOI:** 10.3389/fmed.2022.828695

**Published:** 2022-03-30

**Authors:** Jef Vanhamel, Thijs Reyniers, Edwin Wouters, Josefien van Olmen, Thibaut Vanbaelen, Christiana Nöstlinger, Heleen Van Mieghem, Ella Van Landeghem, Anke Rotsaert, Marie Laga, Bea Vuylsteke

**Affiliations:** ^1^Department of Public Health, Institute of Tropical Medicine, Antwerp, Belgium; ^2^Department of Sociology, University of Antwerp, Antwerp, Belgium; ^3^Department of Family Medicine and Population Health, University of Antwerp, Antwerp, Belgium; ^4^Department of Clinical Sciences, Institute of Tropical Medicine, Antwerp, Belgium

**Keywords:** pre-exposure prophylaxis, HIV prevention, family physicians, primary care, service delivery

## Abstract

**Introduction:**

In Belgium, the provision of pre-exposure prophylaxis (PrEP) for HIV prevention is centralized in specialized HIV clinics. Engaging family physicians in PrEP care could help scale-up its delivery and reach underserved populations. The objective of this study was to gain insight into family physicians' self-perceived roles in providing PrEP.

**Methods:**

We conducted 16 online group discussions with a total of 105 Flemish family physicians, between November 2020 and February 2021. A brief online questionnaire assessed their socio-demographics and experience with sexual health. We analyzed verbatim transcribed data using a grounded theory approach.

**Results:**

Despite limited awareness and experience, participants reported a high willingness to be more actively involved in PrEP care. Four potential roles for the family physician in PrEP care were identified: acting as low-threshold entry point for advice; opportunistic case finding of PrEP candidates; initiating appropriate care for PrEP-eligible clients; and ensuring high-quality follow-up care for PrEP users. Participants framed each of these roles within their current activities and responsibilities as primary care providers. Yet, participants differed in their views on the concrete operationalization of these roles, and in the extent of their involvement in PrEP. Particular challenges were a lack of experience with antiretrovirals, perceived limited exposure to clients at high HIV risk, and a lack of expertise and resources to conduct time-intensive risk assessments and counseling related to PrEP.

**Conclusion:**

Belgian family physicians demonstrated a keen willingness to be involved in PrEP care, but had differing views on the practical implementation into their practices. Providing tailored training on sexual health and PrEP, and investing in collaboration between primary and secondary care, could optimize the integration of PrEP in the primary care practice.

## Introduction

Oral pre-exposure prophylaxis (PrEP) is a very effective HIV prevention strategy, carrying the potential to have a significant impact on the HIV epidemic globally (1–3). Despite a growing number of countries implementing PrEP programs in routine health care services, it was estimated that about 1.3 million persons had initiated PrEP by mid-2021 (1). This estimate still falls short of the three million target set for 2020 by UNAIDS as part of its fast-track strategy to end AIDS as a public health threat by 2030 (2, 3). The slow scale-up of this novel HIV prevention method suggests that some individuals experience barriers toward its uptake (4, 5). De-centralized service delivery models that bring PrEP closer to communities at risk of HIV infection, may reduce some of these barriers (6, 7). In this regard, the involvement of primary care practitioners (PCP) in PrEP care may be a pivotal strategy to scale-up its delivery (8). Their typical ‘point of entry’ status and focus on a holistic approach might facilitate both access to PrEP and the engagement of clients in follow-up care (9, 10).

Adopting PrEP in the primary care practice has, however, not always been straightforward. Early studies conducted in the U.S. reported a lack of PrEP awareness among PCPs and insufficient knowledge of clinical guidelines (11). Other studies showed PCPs having skeptical attitudes toward PrEP, fueled by concerns about side effects, drug resistance, or a potential surge in the incidence of sexually transmitted infections (STI) as a consequence of reduced condom use (11, 12). HIV specialists' perceived low attendance to PrEP-eligible clients combined with PCPs' relative lack of experience with prescribing antiretrovirals led to a “purview paradox,” whereby neither HIV specialists nor PCPs initially perceived PrEP to fall within their scope of clinical activities (13, 14). More recent studies suggest that the willingness to prescribe PrEP increases as experience with PrEP grows (12, 15, 16). Nevertheless, it has been repeatedly shown that many PCPs experience barriers to proactively discuss sexual health with their clients, potentially impacting on their ability to identify suitable PrEP candidates (9, 17–20).

In 2017, Belgian health authorities approved the use of publicly funded PrEP by HIV-negative individuals who meet the eligibility criteria (see Supplementary Material 1). The delivery of PrEP in Belgium is organized through a centralized system of 12 specialized HIV Reference Centers (HRCs). These HRCs are usually embedded in secondary or tertiary health facilities, located in urban or semi-urban areas, and were initially founded to provide multidisciplinary care for people living with HIV. Between 2017 and 2019, 4,071 individuals have been enrolled in PrEP care through these HRCs (21).

Family physicians (FPs) are the cornerstone of primary health care in Belgium, with a key role both in curative and preventive care (22). Yet, FPs currently have no formal role in the provision of PrEP in Belgium. That is, current policies stipulate that the yearly reimbursement request to the national health insurance system should be filed by a specialist physician working in an HRC in order to deliver the first PrEP prescription (23). Family physicians could, in theory, prescribe refills for PrEP once the yearly reimbursement has been obtained. Yet, this practice is currently not promoted or incentivized by official health authorities. Engaging FPs in PrEP care might nevertheless provide opportunities to scale-up PrEP services, for instance by facilitating access to groups that are currently not yet reached by specialist services. Notably, almost all (96%) PrEP starters in 2020 fell under the classification of ‘men who have sex with men’ (MSM), while about half of all new diagnoses in Belgium are attributed to heterosexual sex (21). Surveys conducted in 2014 and 2017, respectively among people with a sub-Saharan African migrant background and MSM living in Belgium, also found that about one third of both populations (30.3 and 33.2%, respectively) was eligible for PrEP according to Belgian eligibility criteria (see Supplementary Material 1) (24, 25). Wary of their limited generalizability, these data suggest a remaining unmet need for PrEP in Belgium. Moreover, FPs perform half (51%) of all HIV tests in Belgium, and, in 2020, they diagnosed 42% of all new HIV infections (21). These figures illustrate the FP's crucial gatekeeping position in primary care, as well as opportunities for their engagement in PrEP care to complement current HIV prevention strategies in Belgium.

In this study, we aimed to gain an in-depth and contextualized understanding of how Belgian FPs view their role in providing PrEP care. We additionally explored opportunities to increase FPs engagement in PrEP, aiming to provide recommendations for future practice and policy. Little research in Europe has focused on PrEP from a primary care perspective, with a particular absence of qualitative studies concerned with FPs' views on the integration of PrEP into primary care. Such studies could provide rich and useful insights into the daily reality of health workers' practice, and help clarify context-specific barriers and facilitators toward the adoption of new interventions, such as PrEP (26).

## Methods

### Study Design

We conducted an explorative qualitative study, using group discussions as data collection technique. We considered group discussions most appropriate to elicit and understand FPs' perspectives, given its process of sharing meaningful experiences and comparing practices between participants (27). Since we conducted this research with already established groups of individuals, we prefer to use the term “group discussion” over the more specific term “focus group discussion” (28). We assembled a multidisciplinary research team, consisting of both clinical researchers (including FPs and physicians with clinical PrEP experience), and public health researchers with a medical or social science background. All members of the research team were involved in the entire research process.

### Study Participants and Recruitment

In Belgium, FPs must be affiliated with local peer groups for continuous medical education, and attend at least two out of four scheduled meetings per year. To reduce the threshold for participation to this study, we organized our group discussions during meetings of such existing peer groups. The purpose of the session was framed as two-fold, namely providing education on PrEP, and collecting data to capture FPs' perspectives on their potential role in PrEP care. Via e-mail, we invited the coordinators of all peer groups in Flanders to participate in this study. Once the coordinator accepted the invitation on behalf of the group, all individual physicians affiliated to that group were informed about the study by the peer group coordinator using a premade information sheet. Participants were instructed to contact the researcher directly via e-mail to provide their informed consent to participate in the study. We held group discussions with all available peer groups that agreed to participate during the planned study period (November 2020-February 2021). Participants were not reimbursed for their participation in this study.

### Data Collection

Before participating in the discussion, FPs were asked to complete a short online questionnaire on socio-demographics and experiences with delivering sexual health care, including PrEP. Due to COVID-19, all group discussions were conducted using web-conferencing technology. Discussions lasted between 80 and 100 min, and we aimed to include 5 up to 10 participants per group. Larger groups of FPs were divided into smaller groups to optimize discussion dynamics. They were moderated by two members of the research team, mostly a social science researcher in tandem with a medical PrEP expert. Both were trained in qualitative research.

The topic guide was designed to facilitate discussions on FPs' involvement in PrEP care, using open-ended questions, case vignettes and statements. The vignettes introduced a fictional client, following a hypothetical care trajectory in a typical primary care setting (see Supplementary Material 3). Participants were then prompted to identify an HIV prevention need in the cases, and discuss their role as FP in answering that need, including PrEP provision and follow-up. The case scenarios as outlined in the vignettes were written by the first author and reviewed by all members of the research team for clarity and clinical accuracy. Additionally, we used statements (e.g., “It is the FP's role to identify clients that could benefit of PrEP.”) to further stimulate reflection and discussion. To investigate how prior PrEP knowledge and experience influenced participants' perceptions and attitudes, statements and case scenarios were initially presented without additional background information on PrEP. As we anticipated that prior PrEP-related knowledge and experience was likely to be low, we paused at dedicated time points during the group discussion (i.e., as specified in the topic guide) to gradually present more information on PrEP and relevant care aspects. Hence, as the case scenarios unfolded, we allowed participants to develop and express informed opinions.

Throughout the data collection phase, we used preliminary insights from debriefings to slightly adapt and improve the topic guide, as per the iterative nature of qualitative research. In later discussion groups, where sessions did not provide any new insights into the role of FPs in PrEP care (i.e., data saturation), we focused more on strategies and tools to support FPs in their future engagement in PrEP care (29).

### Data Analysis

All group discussions were audio- and video-recorded with participants' consent. We analyzed field notes and verbatim transcribed data in QSR Nvivo (release 1.3, March 2020), using a grounded theory approach (30). The first and second author (JV and TR, respectively) developed an initial data-driven codebook based on a first reading and open coding of the transcripts. They then discussed this initial coding framework within the research team. As a next step, the first author re-read and re-coded the transcripts and modified the codebook in close collaboration with the second author. This data-driven descriptive codebook formed the basis for further axial and selective coding to reveal and describe underlying relationships between the identified themes and categories. Results of the coding processes were discussed regularly within the research team to ensure consistency and validity.

## Results

We conducted 16 group discussions with a total of 105 participants. The size of the individual discussion groups ranged from 4 up to 13 participants. There were more female (56.2%) than male (43.8%) participants, and about half of them were between 31 and 51 years old (49.5%). Additional socio-demographics and experiences related to sexual health are shown in Table 1. More information on the composition of each group can be found in Supplementary Material 4.

**Table 1:** Socio-demographics and reported sexual health care experience of FGD participants.

	***N***	**%**
Participants	105	100
Sex		
Male	46	43.81
Female	59	56.19
Age group		
20–30yrs	21	20.00
31–40yrs	32	30.48
41–50yrs	20	19.05
51–60yrs	16	15.24
>60yrs	16	15.24
Years of experience as FP		
<5yrs	20	19.05
5–10yrs	28	26.67
10–20yrs	18	17.14
>20yrs	39	37.14
Estimated weekly patient load		
<10	2	1.90
10–50	14	13.33
51–100	62	59.05
>100	27	25.71
Type of practice		
Solo	15	13.51
Group (mono-/multidisciplinary)	79	71.17
Group (multidisciplinary, forfetary)	8	7.21
Other	15	8.11
Number of HIV patients in follow-up		
None	37	35.24
1–5	65	61.90
5–10	3	2.86
>10	0	0
HIV testing frequency		
Never	2	1.90
Rarely	5	4.76
Few times per year	58	55.24
Few times per month	35	33.33
Few times per week	5	4.76
Last performed sexual history taking		
Never done	3	2.86
A few years ago	6	5.71
A few months ago	20	19.05
Less than a month ago	44	41.90
Less than a week ago	32	30.48
Ever received a question from clients about PrEP		
Yes	44	41.90
No	61	58.10
PrEP experience		
No clients on PrEP to my knowledge	63	56.76
Clients on PrEP but no role in care	37	33.33
Clients on PrEP and role in follow-up	5	4.50
Ever prescribed PrEP	2	1.80
Other	4	3.60

**Figure 1:**
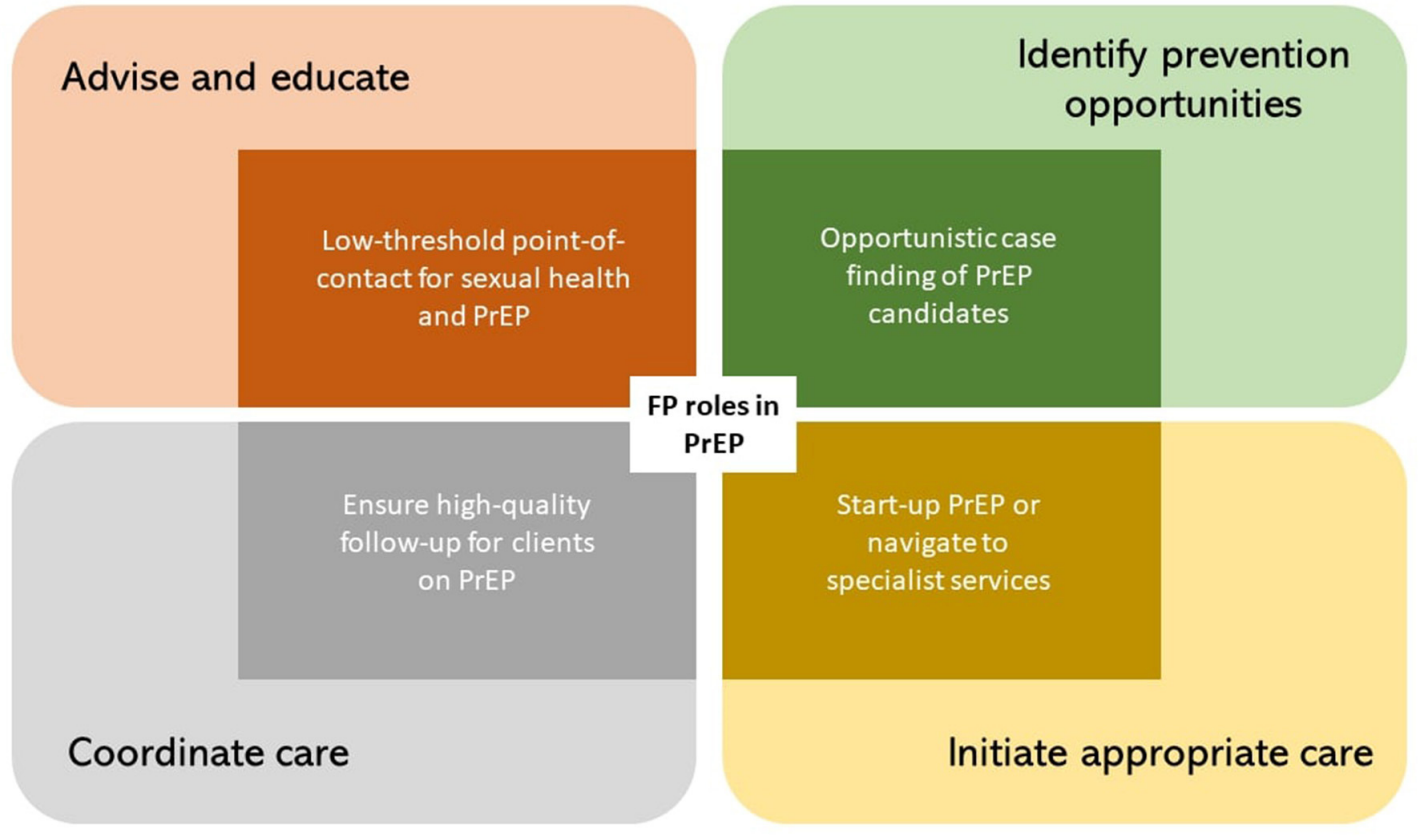
Four self-perceived roles of the FP in PrEP (inner rectangles), framed within their respective more generic FP roles (outer rectangles).

Despite reporting limited current experience with PrEP (see Table 1), participants identified four potential roles for them in PrEP care: (1) acting as low-threshold point-of-contact for advice, (2) opportunistic case finding of PrEP candidates, (3) initiating appropriate care for PrEP-eligible clients, and (4) ensuring high-quality follow-up for clients on PrEP. They framed these roles within a set of broader roles and core responsibilities that they already took up in primary care (see Figure 1). Additionally, we elicited participants' self-reported barriers and facilitators toward the adoption of these PrEP-specific roles into their daily practice as FP.

Below, we discuss each role in PrEP care more in detail. We describe how participants perceived these roles to be in line with general responsibilities of being an FP, and expand on anticipated barriers and facilitators toward their adoption in the primary care setting.

### Family Physicians as Advisors and Educators: A Low-Threshold Point-of-Contact for Sexual Health, Including PrEP

Participants in our study unequivocally agreed that a key role of the FP was to serve as a low-threshold point-of-contact for questions, concerns and advice in all matters of health, including sexual health:

“*We are doing so many things around health, and sexual health is a part of the package we offer, for sure. I don't really see where people would otherwise go with their questions. We are the first point of contact for them anyway.”*

[GD number 14, participant 905: male, less than five years active as FP]

Upon presenting participants with a case demonstrating a high HIV prevention need (i.e., a cis-gender man with a history of condomless anal sex with multiple male partners over the past 6 months), most participants suggested promoting condom use and HIV and STI testing. Only few FPs proactively mentioned PrEP as a possible prevention option. Main barriers reported were a lack of awareness of PrEP and low familiarity with PrEP-related care. Even though nearly half (41.90%) of participants had already received questions from clients about PrEP (see Table 1), qualitative data showed that most FPs were not sure how to answer these questions.

“*My first contact with PrEP was a young man, about 20 years old, who came with a printed internet sheet to request this product. I said to myself “what is this?”. I had never heard of it before and did not know what he was asking for. I referred him to the clinic where I refer my HIV positive clients to, because I assumed they would be able to help him there. I have not seen him back since.”*

[GD number 14, participant 904: female, 5-10 years active as FP]

Initial skepticism toward PrEP emerged as an additional barrier, with some participants calling it an ‘exceptional’ intervention or a last resort when more familiar prevention options (e.g., condoms) had failed. A frequently coined argument was the fear that PrEP would result in an increased incidence of other STIs due to potentially reduced condom use. Several other participants countered this argument with a pragmatic attitude, framing PrEP as a complementary prevention option contributing to the concept of “safe sex”. As familiarity with PrEP increased over the course of the session, participants' interest grew, and attitudes toward their involvement in PrEP care often shifted as the discussion progressed:

“*…I still have some moral difficulties, because there are other ways to protect yourself [from HIV] that cost society less, and that are as effective. But, uhm, on the other hand… I don't know, I also agree with what [participant 212] just said, that we don't need to judge people's behavior. Maybe I just need to think about this really well, and then I might have a different opinion.”*

[GD number 3, participant 208: female, 5-10 years active as FP]

Participants mentioned that dedicating specific attention to PrEP in FP training could help to increase FPs' awareness and knowledge of PrEP. Being able to rely on FP-adapted and accessible evidence-based information on PrEP, and embedding PrEP into a comprehensive sexual health approach with specific attention to other STIs, were generally mentioned as preconditions to deliver adequate advice.

“*Yes, I think we do need to know very well what this [PrEP] is about. We will have to be able to give information, if clients ask for it. We need to have a neutral position about this and provide all options. But I think follow-up and counseling on sexually transmitted infections will be essential.”*

[GD number 1, participant 101: female, more than 20 years active as FP]

### Family Physicians as Identifiers of Prevention Opportunities: Opportunistic Case Finding of PrEP Candidates

Participants discussed their role in proactively identifying opportunities for prevention, including for PrEP. This role was often framed within the inherent holistic approach applied in primary care, offering enhanced insights into clients' lived environment and the contextual factors that co-determine health outcomes:

“*We often have a good view on the circumstances people live in or what their family situation is like. So, even if it were just to keep it [PrEP] actively in mind, I think that must be possible.”*

[GD number 5, participant 401: female, more than 20 years active as FP]

Participants anticipated several barriers for identifying potential PrEP candidates in the FP setting. Finding suitable occasions for conducting sexual risk assessments was deemed difficult, especially in the absence of an entry point to talk about sex. Participants explained that clients could perceive a proactive approach as too intrusive, jeopardizing the trust relationship. A lack of time or skills to perform comprehensive sexual health assessments were mentioned as additional barriers:

“*Once you open this conversation, you also have to feel confident and have the knowledge and skills and… yeah, just feeling at ease with it. Because inquiring about sexual behavior just for the sake of it, I mean that's all fine but then you also need to be prepared to respond to the patient's need and so on.”*

[GD number 4, participant 305: female, 5-10 years active as FP]

Not all participants were convinced of the cost-benefit balance of integrating universal screening for PrEP eligibility into routine practice. There may not be sufficient time to cover this subject during the time span of a standard consultation due to competing priorities. Also, many FPs, especially those practicing in rural or semi-urban areas, considered the need for PrEP among their clients to be too low to make this investment:

“*In my 25-year experience, I have known two HIV-positive clients, in a small rural community. I think it will not be a big group of people here to which we can recommend this [PrEP]. Of course, there are always some people, often young people, who can be seen as more promiscuous and where we can keep this in mind. But actively screening for it in this population will not be worth all the work.”*

[GD number 16, participant 812: male, more than 20 years active as FP]

In response, participants suggested a pragmatic approach for finding PrEP candidates in primary care. Opportunities included a client request for STI screening, an STI diagnosis, a request for a general check-up, as part of contraceptive counseling, and when discussing potential effects of medication on libido. Participants suggested to maximize such opportunities via the integration of PrEP eligibility screening into existing guidelines for FPs. This could be achieved though incorporating automated pop-ups in electronic records, to remind FPs to screen for PrEP eligibility when, for instance, requesting an STI test.

### Family Physicians as Initiators of Care: Starting-Up PrEP or Referring Clients to Appropriate Services for PrEP

We discussed a third potential role, related to initiating clients on PrEP. Participants linked this role to their responsibility in navigating clients through the health system to make sure their needs are met. An FP referral could also lower the threshold to seek specialist care, as exemplified by this FP:

“*Like we refer people with specific problems to, let's say, a cardiologist, we have to guide these people [PrEP candidates] in the same way. And I think, indeed, it will also make it easier for them [clients] to go and see an unknown specialist when I referred them.”*

[GD number 4, participant 302: male, 10-20 years active as FP]

Most FPs reported not feeling confident enough to deliver the first PrEP prescription themselves, due to a lack of experience with antiretroviral drugs or the delivery of associated care. Many participants recognized that PrEP care entailed more than merely prescribing pills. They specifically mentioned lacking support and skills in counseling techniques to safely initiate PrEP. Additionally, mainly FPs practicing outside urban areas worried that attending to a low number of clients at high HIV risk would not allow them to build and/or maintain quality of care. In such cases, participants preferred to refer clients to specialized care to initiate PrEP.

“*I think FPs' knowledge about PrEP is still insufficient, and the question is whether, even if we are trained to provide PrEP, we will be exposed enough to clients who need it, in order not to lose newly acquired expertise. I will remember that this exists, I will guide patients in finding the appropriate care, but I think I will forget how to initiate it myself in 5 years.”*

[GD number 12, participant 818: female, 10-20 years active as FP]

A smaller group of FPs reported that they would feel comfortable in putting clients on PrEP, provided that clear and tailored guidelines were available. These participants perceived medical tasks related to PrEP care as rather straightforward, they had a personal interest in sexual health, or a particular commitment to lower the threshold for clients facing too many barriers to access specialized services:

“*I am thinking of a young female client, who engages in sex work in order to buy drugs, who would never go to the city to get PrEP. I think both options [FP and specialist services] are needed. Some clients might want to bypass their FP for this [PrEP], for others that system is too complicated, and for them we can jump in.”*

[GD number 11, participant 901: female, more than 20 years active as FP]

Participants proposed interactive training sessions to increase FPs' abilities in proactively initiating PrEP care. They suggested that these trainings also need to cover skills in sexual health counseling adapted to the needs of populations who could benefit the most of PrEP (e.g., MSM, sex workers or transgender people at high risk for HIV acquisition).

### Family Physicians as Coordinators of Care: Ensuring High-Quality Follow-Up for Clients on PrEP

A last discussed role related to coordinating care for PrEP users, which fitted FPs' commitment to person-centered care. Notably, participating in the follow-up of clients on PrEP contributed to the broad gaze that is typical of the primary care provider, with attention for different aspects related to the health status of PrEP users:

“*I think the biggest advantage for me is that we know people in their whole context. I am now thinking of a patient who has [sexual] risk contacts whenever he does binge-drinking, which results from a traumatic life experience. It is a very narrow approach to only focus on PrEP in such case. I would set goals together with him on multiple domains.”*

[GD number 6, participant 413: female, 10-20 years active as FP]

Participants perceived their involvement in PrEP follow-up as beneficial because it offered an opportunity to familiarize with PrEP. Additionally, FPs deemed participating in PrEP follow-up valuable to improve their confidence in addressing issues related to sexual health, and contribute to a better FP-client relationship. The low perceived complexity of clinical tasks encompassing PrEP follow-up added to the feasibility of incorporating these aspects into the FP practice. Some participants even saw it as a first step toward gradually taking up more responsibilities in PrEP care in the future.

“*I think once you are more familiar with what it all entails, like with any new medication, in the long run I would feel like ‘I've got it now’, and I could do this myself. Like with contraception it was the same way, we have also learned how to do that by now.”*

[GD number 9, participant 510: female, less than 5 years active as FP]

Participants reported several challenges for safeguarding quality of care, which included suboptimal communication and collaboration with HIV specialists, and difficulties with time management. Also, some participants doubted whether clients would accept being followed-up by their FP for PrEP. For example, when clients have personal ties with the FP, as mentioned by this participant:

“*It can be difficult if you are also the FP of their parents, or of other friends or family members… Okay, we are bound to medical secrecy, but for some people … I can imagine they would feel more comfortable to discuss this with somebody who is more distant, compared to the FP who also treats their grandmother.”*

[GD number 8, participant 501: female, 5-10 years active as FP]

Participants suggested several ways to adopt a proactive role in PrEP follow-up. They proposed maintaining a low-threshold link with specialist care for referral of complex cases, or in case of questions, or to keep track of the latest scientific developments on PrEP. Hence, they preferred a collaboration model for PrEP, with a clear division of roles and responsibilities between specialist physicians (e.g., for starting PrEP) and FP (e.g., for follow-up). Furthermore, participants expressed the need for clear and uniform clinical PrEP guidelines, adapted to the family medicine practice. To their knowledge, such guidelines were now absent.

“*It reminds me a bit of the care trajectories that we have for diabetes, where there is a shared responsibility between us and the specialist physician. The advantage is low-threshold access for patients, and for us being regularly updated by the HIV specialist. That could be a nice collaboration, where patients go, let's say, once a year to the HIV specialist, and in-between they come to us. I would like that.”*

[GD number 7, participant 411: female, less than 5 years active as FP]

## Discussion

In this qualitative study, we explored the perceptions and attitudes of Belgian FPs regarding their role in the service delivery of PrEP for HIV prevention. Despite initial low awareness, participants in our study had a high interest in PrEP. In general, they were willing to be actively involved in PrEP care, albeit with varying degrees of preferred engagement.

Acting as a first entry point for advice on sexual health including PrEP, and referring people who could benefit of PrEP to specialized services, were seen as a minimum degree of involvement for all FPs. Participants framed these roles within the core values of primary care, namely to deliver integrated and holistic care, and to ensure care continuity. Family physicians in our study differed, however, in their views on FPs' engagement in starting clients on PrEP and in providing follow-up care. Where previous quantitative studies mainly explored whether FPs would be willing to prescribe PrEP, our study thus provides a deeper understanding of their preferred degree of involvement in PrEP care (16, 31, 32). One central aspect that may influence such preferences, is preserving an acceptable cost-benefit balance in the FP practice. We found that participants who anticipated not being confronted regularly with a demand for PrEP, perceived the investment of having to re-familiarize with PrEP on limited occasions too much of a burden. This barrier of a lack of demand experienced by some FPs was also reported in a study among PrEP-prescribing FPs in Australia (33). Alternatively, we found that some FPs expressed a particular interest in being able to initiate clients on PrEP without the need for referral. These FPs were often located in settings they perceived as more likely to encounter PrEP-eligible clients (e.g., urban areas). Some of these FPs were committed to offering “one-stop shop” services to clients experiencing too many barriers to access specialized services. Our results thus stress the importance of investing in a PrEP delivery model that allows interested FPs to build sufficient experience. At the same time, strong linkages with PrEP-prescribing practices, such as specialized HIV clinics, have to be built and maintained, for instance to accommodate referrals from FPs less comfortable with prescribing PrEP. This need for collaborative care models for PrEP was also stressed in a recent study among German FPs (34). Such models could help to avoid potential rural-urban disparities in PrEP uptake, as FPs with PrEP discomfort were previously found to cluster in rural areas (35–37).

Previous research has applied a focus group methodology to study provider perspectives on the integration of PrEP in primary care (14). However, we found no examples of studies using a group discussion methodology to unravel the views of family physicians in particular on this topic. In doing so, we were able to study more in-depth the dynamics of participants' views and attitudes toward PrEP when presented with new knowledge on the topic. Consistent with previous studies, we found that FPs' limited awareness and knowledge of PrEP were important barriers toward their perceived involvement in care (11, 16, 34, 38). As FPs learned more about PrEP over the course of the discussion, they grew more comfortable with the idea of being engaged in several related care aspects, as shown in quantitative studies (16, 39). Interestingly, we watched this dynamic unfold over the course of interactive discussion groups. Our study thus suggests the added value of expert-led interactive sessions with peers to respond to FPs' questions or immediate concerns about PrEP. This likely contributed to their increased willingness to be involved in care toward the end of the session. Additionally, besides a knowledge gap, FPs in our study also reported lacking experience with providing PrEP care. This could at least partly be explained by the centralization of PrEP care in specialized HIV Reference Centers in Belgium, with no formalized role for FPs (23). Additional training, together with a policy framework that allows inclusion of FPs in care for PrEP clients, is therefore a prerequisite to increase FPs' engagement in PrEP care in Belgium.

This study also revealed some implementation challenges that require attention. For instance, participants in our study were generally not in favor of implementing universal screening practices for PrEP eligibility into their practices. Participants perceived that conflicting priorities leave less time to be spent on primary prevention activities. Also, participants were afraid that providing unsolicited preventive advice could have detrimental effects on the trust relationship with clients, in particular within the sexual health domain, where some participants feared to come across as intrusive or inappropriate. These arguments are not new, as previous studies have described the challenge of prioritizing primary prevention, such as PrEP, in primary care (40–42). When delivering care within the context and (time) constraints of a typical clinical care visit, FPs usually need to decide which issues will require their immediate attention. In non-preventive care visits, it is therefore more likely that primary prevention will be either omitted or deferred (42). Importantly, when FPs do arrive at discussing prevention, they need to be able to rely on a readily available evidence base. However, previous studies showed how HIV risk assessments by Belgian FPs were often largely based on their personal assumptions, rather than on evidence-based criteria (20, 43). This is consistent with our findings, as many FPs assumed their clients would not be at substantial risk of HIV. Yet, many of them did not conduct regular risk assessments to objectify this risk. Besides issues of time and a lack of guidelines, this finding could also be linked to FPs' discomfort to inquire sexual behavior in clients. As shown in previous studies, FPs often lacked the confidence to discuss sexual health in the absence of an obvious entry point (20, 44, 45). If unaddressed, these practical challenges could undermine FPs' potential to identify suitable PrEP candidates among their clients.

### Recommendations for Future Practice

Family physicians need to be well-equipped and prepared to deal with the demand for PrEP that they encounter in their practices. Since every primary care practice operates in a unique local context, there is a need for tailored support, including options for collaboration with more PrEP-experienced physicians. The development of a (national) PrEP implementation guidance, adapted to the FP practice, could be a first step to outline possible roles and responsibilities for FPs and specialists in PrEP care.

Training on PrEP should not only focus on clinical care aspects, but also contain elements of sexual health counseling sensitive to the needs of potential PrEP users, such as men and transgender people having sex with men (46, 47). Our study shows that interactive sessions within a continuous medical education framework are an acceptable format to train FPs in this regard. In our research, we experienced the value of combining advice and instant feedback of routinized experts with peer discussions. A recent study from the U.S. also found that PrEP-experienced providers recommended interactive formats for future training initiatives on PrEP (48).

Additional resources and practical re-organization, such as task-shifting of time-intensive PrEP counseling activities to nurse practitioners, might offer a more structural approach to facilitating PrEP implementation in primary care (49). This fits within the current attempts to strengthen the capacity of primary health care in Belgium, for instance by enabling FPs to include nurses in a multidisciplinary primary care practice.

Lastly, support tools should be developed and made available to FPs, so to enhance opportunistic case finding of potential PrEP candidates through their practices. For instance, automated pop-up messages were suggested by participants to help remind them of discussing PrEP when requesting an STI test or while conducting a (sexual) history taking. Such reminders may stimulate the adoption of (rapid) screening for PrEP eligibility as part of routine practice. In order to maximize their success and identify more ‘hidden’ potential PrEP candidates, such tools should be embedded within an overall positive approach to sexual health promotion and HIV prevention (50). This means that providers frame sexual health as an integral aspect of human health and explore possible unmet needs of clients through proactive and non-judgmental communication, even beyond explicit requests from clients (51, 52).

### Recommendations for Future Research

Further implementation research could explore the feasibility and acceptability of particular interventions aimed at increasing FPs' involvement in PrEP care. Also, interventions to foster interdisciplinary collaboration for the provision of sexual health care have to date been insufficiently reported. In this regard, much could be learned from experiences in collaborations to tackle other health problems, such as the development of interprofessional care plans for the management of diabetes (53, 54). Moreover, there is a need to explore specialist physicians' perceptions toward the participation of FPs in PrEP care, to inform and guide possible de-centralization pathways for PrEP in Belgium. For instance, studies conducted elsewhere have found that HIV specialists were generally not in favor of FPs taking up more responsibilities in health care domains that they consider as more “specialized” (e.g., sexual health care and care for LGBTQI+ people) (55). Lastly, further research may focus on how urban-rural differences in availability of high-quality sexual health care services may impact on health and wellbeing of sexual and other minority groups.

### Study Limitations

Our sample was based on voluntary participation, and thus a selection bias cannot be excluded. Yet, since local coordinators accepted the invitation on behalf of the entire peer group, it is plausible that also FPs without specific interest in sexual health and PrEP have participated. Moreover, since we worked with pre-existing groups, the absence of purposive sampling might have caused some “within-group” imbalances in terms of age and gender. However, the overall sample reflected a balanced mix of these socio-demographic characteristics. Additionally, socially desirable answers may occur when collecting self-reported data, particularly when working with pre-existing groups that meet regularly. For instance, participants might have been more inclined to report positive attitudes toward FPs' engagement in PrEP care out of fear of negative judgments from their peers. However, this bias might have been mitigated by having trained qualitative researchers as moderators, skilled to create a non-judgmental environment in which non-popular opinions are equally valued. Moreover, the discussions may have been more ‘naturally occurring’ as these pre-existing groups regularly meet to discuss FP-related matters, reducing the barriers for commenting on each other and enabling an environment to build on shared experiences (56). Lastly, due to COVID-19, all discussion groups were held online. This may have come with potential trade-offs regarding data richness compared to face-to-face discussions, as online tools might carry less potential to stimulate lively group discussions or capture non-verbal cues (57, 58).

## Conclusion

This study provided valuable insights into Belgian FPs' self-perceived roles in providing PrEP, showing a high willingness to be actively involved in PrEP care, albeit with different degrees of preferred engagement. We revealed important opportunities for a successful integration of PrEP into primary care, although some implementation barriers still need to be addressed. If we are to scale-up PrEP to have a maximum impact on HIV incidence and sexual health, there is a need for additional, complementary, service delivery options. Our study outlines future directions in how to meaningfully engage FPs in PrEP care, hereby contributing to making PrEP service delivery more responsive to clients' needs.

## Data Availability Statement

The datasets presented in this article are not readily publicly available because they contain information that could compromise the privacy of our research participants. Additional data are available from the first author on reasonable request.

## Ethics Statement

The studies involving human participants were reviewed and approved by Institutional Review Board of the Institute of Tropical Medicine Antwerp. All participants were required to provide verbal informed consent to participate.

## Author Contributions

JV: writing of the protocol and developing the methodology, data collection and investigation, lead in formal analysis, project administration, and writing of the original draft and subsequent versions. TR: conceptualization of the study, writing of the protocol and co-developing methodology, data collection, participation in data analysis and interpretation, review of original draft, and co-writing subsequent versions. EW, JO, and CN: writing of the protocol, data collection, participation in data interpretation, review of original draft, and co-writing subsequent versions. TV, EL, and HM: data collection, review of original draft, and co-writing subsequent versions. AR: writing of the protocol, review of original draft, and co-writing subsequent versions. ML: supervision, funding acquisition, review of original draft, and co-writing subsequent versions. BV: supervision, funding acquisition, writing of the protocol, data collection, participation in data interpretation, review of original draft, and co-writing subsequent versions. All authors contributed to the article and approved the submitted version.

## Funding

This study was conducted within the research project PROMISE: optimize PrEP to maximize impact, funded by the Flemish Research Foundation (FWO) as Strategic Basic Research project (SBO) (S004919N). The funder had no role in the study design, data collection, analysis and interpretation of data, or in writing the manuscript.

## Conflict of Interest

The authors declare that the research was conducted in the absence of any commercial or financial relationships that could be construed as a potential conflict of interest.

## Publisher's Note

All claims expressed in this article are solely those of the authors and do not necessarily represent those of their affiliated organizations, or those of the publisher, the editors and the reviewers. Any product that may be evaluated in this article, or claim that may be made by its manufacturer, is not guaranteed or endorsed by the publisher.
