# Supplementary material for: How Do Family Physicians Perceive Their Role in Providing Pre-exposure Prophylaxis for HIV Prevention?–An Online Qualitative Study in Flanders, Belgium
Source: Front Med (Lausanne). 2022 Mar 30;9:828695. doi: 10.3389/fmed.2022.828695 (PMC9005841; doi:10.3389/fmed.2022.828695)
Supplement: Supplementary file 1 [file Table_1.DOCX]

Supplementary Material

# Supplementary material 1: Reimbursement criteria for publicly funded PrEP in Belgium

| FTC/TDF (emtricitabine/tenofovir disoproxil fumarate) in Pre-Exposure Prophylaxis (PrEP) for HIV prevention. Reimbursement criteria in Belgium :  - HIV-negative status by testing - AND high risk of HIV acquisition, determined by having at least one of the following risk factors: | |
| --- | --- |
| Men having sex with men | Others at high risk of HIV |
| Who have had condomless anal sex with at least 2 partners in the last 6 months.  - Who had >1 STD (syphilis, chlamydia, gonnorrhea or a primary infection with hepatitis B or C) in the last 12 months - Who needed PEP(post-exposure prophylaxis) >1 time in the last 12 months - Who use psycho-active substances during sex | People who inject drugs and share needles  - Individuals exposed to unprotected sex in their profession (sex work) - Individuals who are in general being exposed to unprotected sex with a high risk of HIV acquisition - Partners of HIV-positive individuals not virally suppressed (during ART initiation or no viral suppression despite ART treatment) |
