# Supplementary material for: How Do Family Physicians Perceive Their Role in Providing Pre-exposure Prophylaxis for HIV Prevention?–An Online Qualitative Study in Flanders, Belgium
Source: Front Med (Lausanne). 2022 Mar 30;9:828695. doi: 10.3389/fmed.2022.828695 (PMC9005841; doi:10.3389/fmed.2022.828695)
Supplement: Supplementary file 3 [file Table_3.DOCX]

Supplementary Material

**Supplementary material 3 – Case vignettes.**

| **Case vignette #1 – a client with an unmet HIV prevention need**  Bart is a young man of 22 years old who comes to your consultation. He has a blank medical history and does not normally come to your practice for consultations very often. After some hesitation, he tells you how he recently had a conversation with someone about HIV while visiting a gay bar in the neighborhood. This has made him think a bit, and he now comes to you for advice on what he can do to protect himself better. You never talked to him about sexual health before, and this is the first time you get any information on his sexual identity.  **Discussion**  You conduct a sexual history taking, and this reveals that he identifies as a homosexual. He does not currently have a steady partner, but he regularly has casual sexual intercourse during nightlife. He has both oral and anal sexual contact. He always tries to carry condoms with him, but admits that he has difficulty using them, especially when he has been drinking. In the past 6 months, he had anal sex without a condom at least three times. He was always the receptive (receiving) partner. He has never been much concerned about STDs or HIV, and to his knowledge, has never contracted any of these infections. Now that he has a better understanding of what HIV is, he would like to protect himself better in the future.  **Explanation on PrEP**  PrEP is a combination of two oral antiretroviral drugs (TDF/FTC). Studies have shown that it is a highly effective and safe option for people at increased risk of HIV infection. With good compliance, PrEP reduces the risk of HIV infection by more than 90% and is at least as effective as condom use. PrEP can be taken continuously, on a daily basis, or only during periods of increased risk (‘event-driven’). PrEP complements the current HIV prevention strategy to end the HIV epidemic in Belgium. PrEP should always be used in combination with other existing preventive measures, as PrEP only protects against HIV and not against other STDs. A list of reimbursement criteria that a person has to meet in order to be eligible for PrEP has been drawn up. In addition to a negative HIV test, these include a number of behavioural indicators that may indicate an increased risk of HIV.  **Explanation continued**  In Belgium, it is currently not possible for FPs to initiate clients on PrEP. In order to obtain reimbursement, patients must first make an appointment with an accredited HIV reference centre. An HIV specialist then carries out the necessary screening, which include: a blood test with serum creatinine and HIV, hepatitis B/C and syphilis serology. In addition, protein and glucose are checked on a urine dipstick and patients are screened for chlamydia and gonorrhoea by means of a throat and rectal swab, and a urine PCR. If there are no contraindications, the HIV specialist then delivers the first prescription for PrEP together with the certificate for reimbursement. Finally, counseling in correct PrEP use and the importance of adherence is provided.  **Discussion** |
| --- |

| **Case vignette #2 – a request for participating in PrEP follow-up care**  John is a 41-year-old homosexual man who has been a patient of yours for about 15 years. In the past, he sometimes experienced periods of depression and he has a history of some STDs, namely a recurrent herpes simplex infection and a gonorrhoeae uretritis.  6 months ago, John went to see an HIV specialist at his own initiative, at an HIV clinic about 40 km from your practice, with the request to start on PrEP. As he met the reimbursement criteria and no contraindications were found, the HIV doctor started him on PrEP. Given the long distance to the HRC, John asked the HIV doctor about the possibility of being followed up by his GP instead of the HRC. Today you get a call from the HRC and the HIV-doctor asks you if you would be able to assist in monitoring John.  **Discussion**  **Explanation**  The current guidelines for the follow-up of PrEP users state that a 3-monthly follow-up consultation is indicated. HIV seroconversion is excluded by means of an HIV serology test. Possible side effects of PrEP are investigated, and patients are counseled in compliance and sexual health. Patients are screened for STDs by means of syphilis serology and a urine PCR + throat and rectal swab for chlamydia and gonorrhoea. The kidney function of PrEP users is checked at least annually (or sooner if additional risk factors are present). If reimbursement for PrEP is in order (through an annual certificate through an HIV specialist), the prescription for PrEP can be renewed by any FP.  **Discussion** |
| --- |
