# Supplementary material for: How Do Family Physicians Perceive Their Role in Providing Pre-exposure Prophylaxis for HIV Prevention?–An Online Qualitative Study in Flanders, Belgium
Source: Front Med (Lausanne). 2022 Mar 30;9:828695. doi: 10.3389/fmed.2022.828695 (PMC9005841; doi:10.3389/fmed.2022.828695)
Supplement: Supplementary file 4 [file Table_4.DOCX]

Supplementary Material

**Supplementary material 4 – Composition of the different groups.**
